# The New Anæsthetic

**Published:** 1880-04

**Authors:** 


The New Anæsthetic.—The Bromide of Ethyl (or Hydrobromic Ether). Dr. R. J. Levis gives the results of his observations as to the anæsthetic properties of this drug. It is characterized by rapidity of action and quick recovery from its effects.

It does not influence the circulation to any marked extent, nor is there the same danger from cerebral anæmia and fatal syncope as seen when chloroform is used. In its effects on the respiration, bromide of ethyl resembles ether more than chloroform, producing only ordinary anæsthetic sleep. Nausea and vomiting are less frequent than with other anæsthetics. Its odor resembles somewhat that of chloroform, although it is less agreeable. It also possesses an advantage over chloroform in being entirely eliminated through the lungs. There is less tendency to struggle and to general excitement, when the bromide is used, than when the other anæsthetics mentioned are employed. Anæsthesia is generally produced by this drug in from two to three minutes, and the quantity necessary to produce this result, is from one to eleven drahms.—*N. Y. Med. Rec.* (*Reprint from Phila. Med. Times, Jan.* 17, 1880.)

---

*Oil of Encalyptus an Innocuous substitute for Carbolic Acid.*–Schulz, of Bonn, has found that oleum eucalypti is devoid of all toxic action. This oil appears to combine the various advantages of carbolic acid without sharing its dangerous properties. It was found to have powerful anti-septic qualities ; was readily soluble in alcohol and oil, and mixed well with parassine ; besides it had an agreeable odor.—*Centralbl. fur Chir. No.* 4, 1880.

